# Subplasma membrane Ca^2+^ signals

**DOI:** 10.1002/iub.1032

**Published:** 2012-06-01

**Authors:** John G McCarron, Susan Chalmers, Marnie L Olson, John M Girkin

**Affiliations:** 1 Strathclyde Institute of Pharmacy and Biomedical Sciences27 Taylor StreetUniversity of StrathclydeGlasgow, UK; 2 Centre for Advanced Instrumentation, Department of Physics, Durham UniversitySouth Road, Durham, UK

**Keywords:** Ca^2+^ signaling, calcium mediated signaling, signal transduction

## Abstract

Ca^2+^ may selectively activate various processes in part by the cell's ability to localize changes in the concentration of the ion to specific subcellular sites. Interestingly, these Ca^2+^ signals begin most often at the plasma membrane space so that understanding subplasma membrane signals is central to an appreciation of local signaling. Several experimental procedures have been developed to study Ca^2+^ signals near the plasma membrane, but probably the most prevalent involve the use of fluorescent Ca^2+^ indicators and fall into two general approaches. In the first, the Ca^2+^ indicators themselves are specifically targeted to the subplasma membrane space to measure Ca^2+^ only there. Alternatively, the indicators are allowed to be dispersed throughout the cytoplasm, but the fluorescence emanating from the Ca^2+^ signals at the subplasma membrane space is selectively measured using high resolution imaging procedures. Although the targeted indicators offer an immediate appeal because of selectivity and ease of use, their limited dynamic range and slow response to changes in Ca^2+^ are a shortcoming. Use of targeted indicators is also largely restricted to cultured cells. High resolution imaging applied with rapidly responding small molecule Ca^2+^ indicators can be used in all cells and offers significant improvements in dynamic range and speed of response of the indicator. The approach is technically difficult, however, and realistic calibration of signals is not possible. In this review, a brief overview of local subplasma membrane Ca^2+^ signals and methods for their measurement is provided. © 2012 IUBMB IUBMB Life, 64(7): 573–585, 2012

Increases in the cytoplasmic Ca^2+^ concentration (Ca^2+^ signals) participate in virtually every physiological process (1). At rest, the cytoplasmic free Ca^2+^ concentration ([Ca^2+^]_c_) is maintained at a low (100 nM) value against a constant inward leak from the higher [Ca^2+^] in the extracellular medium (∼2 mM) and in internal stores (several hundred micromolar). A fundamental element of signaling is the controlled entry of the ion into the cytoplasm via channels that are often specific for Ca^2+^ and which respond to extracellular stimulation. This channel activity generates [Ca^2+^] increases of various time courses and locations that are translated to physiological responses. For example, exocytosis is triggered within microseconds by locally high [Ca^2+^] near vesicle release sites (2), contraction occurs over the timescale of hundreds of milliseconds to seconds by a global rise in [Ca^2+^] throughout the cell (a “global” signal), gene expression is regulated in minutes (3) and proliferation over the timescale of days. An understanding of how various Ca^2+^ signals arise and are controlled is, therefore, central to much of biology.

Ca^2+^ signals begin with the opening of one or a few channels. Influx to the cytoplasm occurs at rates of ∼0.6 million Ca^2+^ ions per second per channel (0.2 pA current). The influx generates a significant local concentration gradient near the channel in which [Ca^2+^] declines from ∼10 μM to ∼100 nM over a few hundred nanometers from the plasma membrane (4–10) (Fig. 1). Channel open time is brief (∼1 ms) so that the gradient dissipates rapidly with rates of change in the subplasma membrane space of the order of ∼5000 μM s^−1^, when compared with a maximum of ∼0.5 μM s^−1^ in the bulk cytoplasm (Fig. 1) after a global [Ca^2+^] rise. High local [Ca^2+^] and the rapid rates of change near channels may target processes with rapid Ca^2+^ binding kinetics to selectively activate particular functions (11–15). The high local [Ca^2+^] signals, in turn, may activate neighboring IP_3_ or ryanodine receptor channels (IP_3_R or RyR, respectively) to amplify the local signals or propagate through the cell as global signals with slower but more widespread effects throughout the cell (16–23). The transition of signals from those involving single to multiple channels and from local to global [Ca^2+^] increases creates a multitude of signals with various locations, magnitudes, and time courses (24–27) so that various cellular functions may be selectively activated.

**Figure 1:**
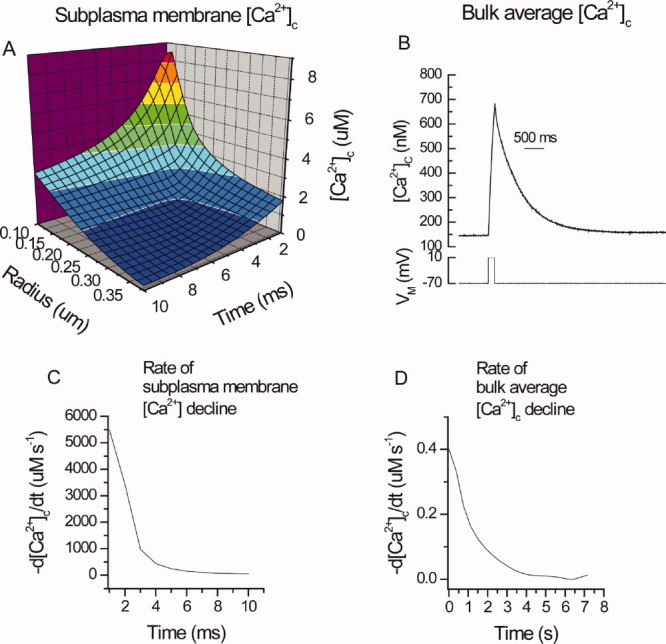
Simulated subplasma membrane [Ca^2+^] changes following the opening of a single Ca^2+^ channel in smooth muscle. A: Microdomains of [Ca^2+^] were produced by the opening of single Ca^2+^ channels. A Ca^2+^ channel opening for on average 2.9 ms with a current of 0.18 pA [39] will increase local [Ca^2+^] near the cytoplasmic aspect of the channel. The magnitude of Ca^2+^ entry (moles) was calculated from the time integrated current: 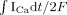 where 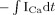
is the total charge entry and F the Faraday constant. The ion will diffuse from the channel randomly creating a hemisphere of increased [Ca^2+^]. The outer radius of the hemisphere was described ((6*Dt*)^1/2^) to express the three dimensional root mean square displacement of an ion through a medium with a diffusion coefficient (*D*) equal to 2.2 × 10^−6^ cm^2^ s^−1^ and *t*, time. A hemisphere volume (not shown) was calculated from the radius at various times and the [Ca^2+^] within determined from the Ca^2+^ moles influx via the single channel divided by the buffer power (100). High [Ca^2+^] (tens of micromolar) within the hemisphere volume exist in the first few milliseconds after channel opening. [Ca^2+^] is a function of both distance from the influx channel and time after channel opening. Note, the [Ca^2+^] was estimated by assuming a uniform distribution of Ca^2+^ in the microdomain (which may not be valid) and Ca^2+^ being in equilibrium with the buffers. The latter may only occur when the association rate constant exceeds 10^8^ M^−1^ s^−1^. (B) The [Ca^2+^]_c_ increase in the bulk cytoplasm in response to entry via voltage-dependent Ca^2+^ channels was substantially less than that of the subplasma membrane; maximum values were in the hundred of nanomolar range. Thus, depolarization of the plasma membrane (−70 mV to +10 mV B lower panel) increased [Ca^2+^]_c_ uniformly throughout the bulk of the cytoplasm. [Ca^2+^]_c_ was restored toward resting levels when the depolarization ended by removal of Ca^2+^ from the cytoplasm by Ca^2+^ pump activity. (C, D) Rate of [Ca^2+^] decline in the subplasma membrane microdomain and bulk cytoplasm following voltage-dependent Ca^2+^ entry. (C) In the subplasma membrane space, the maximum rates of decline were ∼ 5500 μM s^−1^. The data for this figure was derived from Fig 1A. (D) Decline (−d[Ca^2+^]_c_/d*t* μM s^−1^) in the bulk cytoplasm following depolarization-evoked Ca^2+^ entry from the data in Fig 1B. Maximum rates of decline in the bulk cytoplasm approached 0.4 μM s^−1^. Note the scale difference in the abscissae in C, D. (Modified from ref. 4, with permission from Elsevier).

An understanding of Ca^2+^ signaling requires an appreciation of how Ca^2+^ is selectively localized to certain regions of the cell. Interestingly, in this respect, the global Ca^2+^ signals may arise not from the combined activity of channels throughout the cell but from only a small fraction of the overall channel complement, and significantly, often those active channels are largely located close to (IP_3_R and RyR) or on (voltage-dependent Ca^2+^ channels) the plasma membrane (28–30). For example, [Ca^2+^] increases in response to IP_3_-generating agonists may involve <1% of the overall IP_3_R complement with 75% of the active channels being exceedingly close (∼100 nm) to the plasma membrane in SH-SY5Y cells (30). Active RyR are also mainly at the plasma membrane to regulate ion channel activity there (31–37). The positioning of active sarcoplasmic reticulum (SR) Ca^2+^ release channels near the plasma membrane provides a mechanism which may enable agonist activation to target specific types of cellular response, that is, by generating [Ca^2+^] rises in specific regions of the cell. To facilitate this process, some plasma membrane located receptors or signaling cascades co-localize with IP_3_R (38–40) that are positioned near the plasma membrane. For example, while muscarinic and bradykinin receptors each stimulate phopholipase C, only bradykinin receptors coimmunoprecipitate with, and activate, IP_3_R (38) in neurons. The clustering of receptors for neurotransmitters or hormones in certain regions on the plasma membrane (41) may also contribute to the selective targeting of particular responses by providing areas with increased sensitivity to extracellular stimuli. Agonists which operate by releasing Ca^2+^ via IP_3_R may also evoke specific cellular responses by linking to additional signaling pathways to increase flexibility and complexity of the response. Parathyroid hormone stimulates adenylate cyclase to deliver cyclic adenosine monophosphate (cAMP) via “cAMP junctions” to sensitize IP_3_R2 to IP_3_ (39) to enable generation of Ca^2+^ events in specific parts of the cell. Thus, there are direct, specific associations between signaling proteins located at the plasma membrane and those on that part of the SR membrane which is close to the plasma membrane (42). Such associations enable messengers which have the potential to evoke activity throughout the cell (e.g. Ca^2+^), to selectively target particular physiological responses, hence the interest in understanding subplasma membrane Ca^2+^ signaling.

Although voltage-dependent Ca^2+^ channels appear uniformly distributed across the membrane (43), as with IP_3_R, signals arise from a small fraction of the overall channel complement (44) and active channels cluster (45) to facilitate localized specific activation of nearby effectors [Figs. 2 and 3; (see also *44, 46, 47*)]. The clustering enables uniform cell wide activation via depolarization to locally activate selected specific cell processes. In vascular smooth muscle, voltage-dependent Ca^2+^ channels were suggested to be clustered and constitutively active (47). Although our studies show voltage-dependent Ca^2+^ channels are clustered, the channels are controlled by membrane potential (i.e., they lack constitutive activity), a feature which will enable the channels to remain under physiological control (45). Interestingly, during prolonged depolarization, some small sites near the plasma membrane display repetitive Ca^2+^ rises (Fig. 3) (45, 47), which suggests some channels are more readily activated than others and which presumably target specific cellular responses.

**Figure 2:**
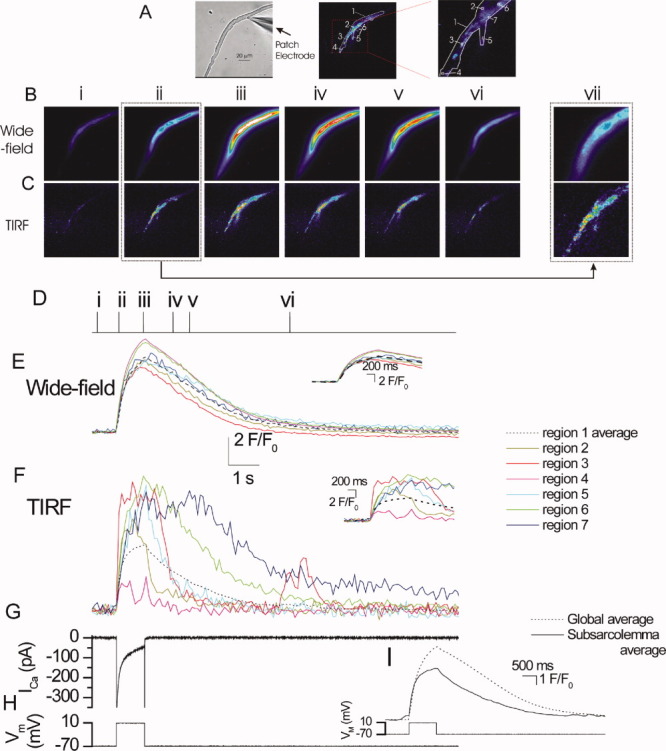
Simultaneous wide-field epi-fluorescence and TIRF [Ca^2+^] measurements in a voltage-clamped single smooth muscle cell. Depolarization (−70 mV to +10 mV; H), activated a voltage-dependent Ca^2+^ current (*I*_Ca_; G) to evoke a rise in [Ca^2+^] (B,C,E,F). The rise in [Ca^2+^] which occurred in the subplasma membrane space (measured by TIRF) (C,F) was more rapid in onset than that seen in the bulk cytoplasm (measured by wide-field epi-fluorescence) (B,E). The [Ca^2+^] images (B, C) are derived from the time points indicated by the corresponding numerals in D. [Ca^2+^] changes in B and C are represented by color; blue low and red/white high [Ca^2+^]. The images (B and C) were taken before (i), during (ii-iii), and after (iv-vi) depolarization and show the resulting [Ca^2+^] changes. Changes in the fluorescence ratio with time (E and F) are derived from 2 × 2 pixel boxes (regions 1-6 in A, middle and right (expanded) panel; drawn at a 3 × 3 pixel size to facilitate visualization) and from a larger region encompassing the entire TIRF region (region 7). The latter was used to obtain an average subplasma membrane and bulk average [Ca^2+^]_c_ increase (I). Significantly, although the [Ca^2+^]_c_ increase which occurred in the bulk cytoplasm (B and E) was approximately uniform and simultaneous throughout the cell, those in subplasma membrane space (C and F) had a wide range of amplitudes and various time courses. Note the spark like events in region 3 toward the end of the recording. (A) left panel show a bright field image of the cell; see also whole cell electrode (right side). Insets in E and F show the rising phase of the transients on an expanded time base. For comparison, I shows the average subplasma membrane and bulk average [Ca^2+^]_c_ rise as measured in region 7 (A, right hand panel). B and C (vii) shows an enlargement of ii to illustrate the localized nature of the rise in [Ca^2+^] in the subplasma membrane space (Reproduced from ref. 45, with permission from Rockefeller University Press).

**Figure 3:**
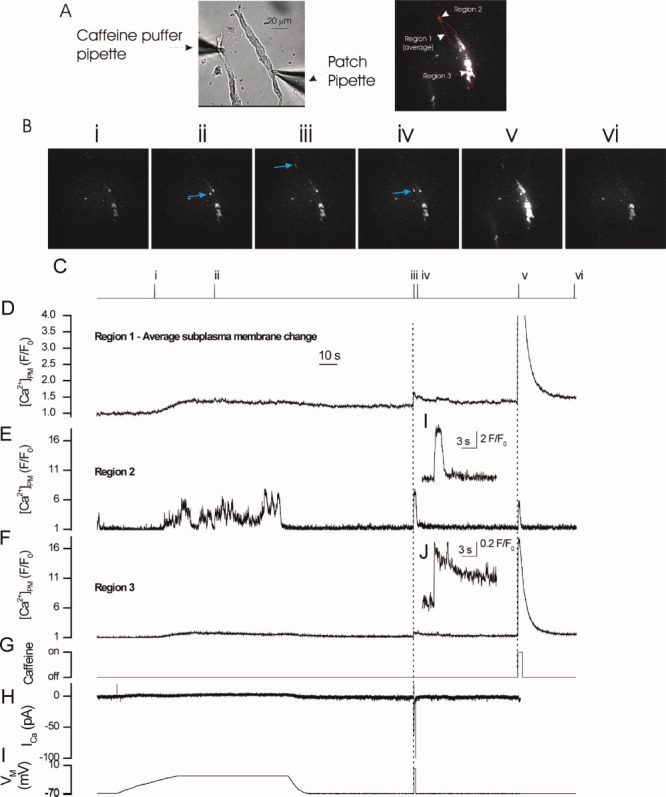
Local subplasma membrane Ca^2+^ transients in response to plasma membrane depolarization and caffeine in a voltage-clamped single colonic myocyte. Gradual depolarization from −70 mV to −20 mV (I) elevated [Ca^2+^] in the subplasma membrane space as measured in TIRF (D-F). The [Ca^2+^] increase was more localized and substantially larger in some regions than others (compare E and F). Two example regions (regions 2 and 3) are shown (panels E and F); localized rises occurred in region 2 but not region 3. Blue arrows in the frames in B (ii-iv) show examples of the localized rises in [Ca^2+^]. The [Ca^2+^] increase which occurred in region 3 (F) was slow and of smaller magnitude than that of region 2 (E). When the membrane potential was restored to −70 mV (I) [Ca^2+^] was returned towards resting levels. A subsequent transient depolarization to 0 mV (H) activated voltage-dependent Ca^2+^ current (*I*_Ca_; H) and increased [Ca^2+^] (D-F). The increase was larger and declined more rapidly in region 2 (D and inset I) than region 3 (E and inset J). Those regions (region 2, E) which showed large responses to depolarization had small responses to RyR activation by caffeine (G). Conversely, a large response (region 3, F) to caffeine (G) occurred in regions with small responses to depolarization. Region 1 (D) is a subplasma membrane average change. Numerals in images (B) correspond to those in C. Changes in the fluorescence ratio with time (D,E,F) are derived from 1 pixel boxes (regions 2 and 3; shown at 3 × 3 pixels to facilitate visualization) and from a larger region the entire TIRF image (region 1) to obtain an average subplasma membrane [Ca^2+^] change (D). The position of the regions from which the transients (D,E,F) were obtained are shown in A (right-hand panel). A bright field image of the cell is shown in (A; left panel); see also whole cell electrode (right side) and puffer pipette which contained caffeine (10 mM; left side) (Reproduced from ref. 45, with permission from Rockefeller University Press).

The growing appreciation of the significance of local signals near the plasma membrane in controlling cell function has led to the development of various methodologies to study subplasma membrane [Ca^2+^]. Some approaches have been based on the sensitivities and activities of Ca^2+^- activated ion channels, such as the Ca^2+^-activated K^+^ channel (*K*_Ca_) to estimate subplasma membrane Ca^2+^ concentrations. Using this approach, concentrations of the order of ∼10 μM have been estimated to occur in the subplasma membrane space (48, 49). In our studies on smooth muscle cells, depolarization-evoked [Ca^2+^]_c_ increases were calculated to raise subplasma membrane [Ca^2+^] to ∼50 μM in the space between the plasma membrane and SR (50). In other experiments, more direct measurements were made using Ca^2+^ indicators. In smooth muscle cells, a fluorescent Ca^2+^ indicator was targetted to the plasma membrane using a lipophilic tail (FFP18) (51). The [Ca^2+^] transients measured in response to plasma membrane depolarization were of the order of micromolar concentrations, though the bulk average [Ca^2+^]_c_ (measured using a cytoplasmic fluorescent Ca^2+^ indicator fura-2) rose by only a few hundred nanomolar (51). In squid giant axons, a microinjected low-affinity aequorin mutant measured “hot spots” of [Ca^2+^] in the range of 200–300μM in the synapse during action potentials (5). Later studies in the large synapses on the calices of Held indicated that, under physiological conditions, fast vesicle release could be explained by smaller (20 μM) (7) increases in subplasma membrane [Ca^2+^]. Together, these early studies were instrumental in highlighting different [Ca^2+^] in the subplasma membrane and bulk cytoplasm and prompted more advanced methodological development to study subplasma membrane [Ca^2+^]. Recently, the two main experimental approaches for visualizing Ca^2+^ signals have been (1) to specifically localize Ca^2+^ indicators to the subplasma membrane space to measure [Ca^2+^] only there or (2) to allow indicators to be dispersed throughout the cytoplasm but to measure the fluorescence emanating from the Ca^2+^ signals only at the subplasma membrane space using high resolution imaging procedures. These procedures will now be briefly reviewed.

## TARGETED Ca^2+^ INDICATORS

In cultured cells, the most prevalent approach to measure Ca^2+^ signals in specific regions of the cell is to use genetically encoded Ca^2+^ indicator proteins (fluorescent or luminescent), which can be targeted to organelles or subcellular locations, for example, the plasma membrane. Probes localized to the inner surface of the plasma membranes have detected Ca^2+^ concentrations in the subplasma membrane space, which significantly exceed those of the bulk cytosol. A chimera of the luminescent protein aequorin measured the mean [Ca^2+^] on the inner surfaceof the plasma membrane of the A7r5 smooth muscle cell line to be ∼1–2 μM at rest (6). The high concentration may reflect the presence of “hot spots” in the vicinity of sporadically opening channels, rather than a homogeneous high [Ca^2+^] in the subplasma membrane space. During store-operated Ca^2+^ influx, in the same cells, subplasma membrane [Ca^2+^]increased to 50 μM (6). Although targeting to the plasma membrane was successful, aequorin has some experimental limitations. The low levels of luminescence and destruction of aequorin by Ca^2+^ complicate analysis of results and, significantly, prevent single cell imaging and high-time resolution comparisons with electrophysiology. To overcome this issue, targeted fluorescent protein based sensors of Ca^2+^ such as the “cameleons”(52) offered some advantages particularly in photostability. However, cameleons are large (650 amino acids), often bigger than the host proteins to which they are fused, which limits their use. In addition, the dynamic response of the cameleons, and indeed other Ca^2+^ sensitive photoproteins, are considerably slower than the physiological response they are used to report. This property may result in the indicator not accurately following the rapid changes in subplasma membrane [Ca^2+^]. In another parallel approach, a calmodulin-based fluorescent protein-based indicator (GCaMP2) was tethered to Na^+^ pumps to report subplasma membrane Ca^2+^ signals. Although the indicator reported changes over timescales of minutes (53), it was again too slow for the milliseconds transients close to the plasma membrane. In an attempt to overcome this speed of response issue, a troponin-based indicator which possesses a relatively fast off-rate was developed (54). Even this indicator is still more than two orders of magnitude slower than typical small molecule Ca^2+^ indictors such as fluo-3 (*k*_on_ of 28.2 μM^−1^ s^−1^ and *k*_off_ of 7.04 s^−1^ (54) compared to 7100 μM^−1^ s^−1^ and 369 s^−1^ for fluo-3 (55). The mean times for TN-XL and fluo-3 to capture (and so detect) a Ca^2+^ change is 35.5 ms (TN-XL) and 0.14 ms (fluo-3)). The mean capture times were calculated from 1/*K*_on_B (56, 57), where B is the free Ca^2+^ binding sites on the indicator and was taken to be 1 μM for each indicator. As shown in Fig. 1, the highly localized subplasma-membrane [Ca^2+^] arising from the opening of a single Ca^2+^ channel declines within 10 ms. Since the mean capture time of the “fast” troponin-based Ca^2+^ indicator (TN-XL) is 35.5 ms, this probe would fail to detect the Ca^2+^ change. Fluo-3 is on the other hand fast enough to detect the Ca^2+^ signal.

Thus, despite continuous improvements, the Ca^2+^ indicator proteins typically have a fairly restricted dynamic range (ratio of signals from Ca^2+^ bound and Ca^2+^ free forms, ∼1–4-fold (54) compared to >100-fold for fluo-3 (manufacturer's literature)) and can be physically large. However, probably most significantly the photoprotein indicators are slow when compared with the rate of change in physiological Ca^2+^ signals beneath the plasma membrane. Together, these features present significant drawbacks in measuring subplasma membrane [Ca^2+^].

Although small synthetic molecule Ca^2+^ indicators, for example, fluo-3 and fura-2 are considerably faster in detecting Ca^2+^ and have wider dynamic ranges than the fluorescent protein Ca^2+^ indicators, targeting of these indicators to specific subcellular sites has been problematic, limiting the indicators' use in reporting subcellular [Ca^2+^]. In some cases, the intrinsic properties of particular indicators reportedly help localize them to certain regions of the cell (e.g., mitochondria) but results have not been universally positive. For example, rhod-2 has been used in some studies to successfully measure mitochondrial matrix [Ca^2+^]. Rhod-2 in its membrane permeant acetoxymethyl (AM) ester form bears a net positive charge which, it is suggested, results in mitochondrial accumulation of the dye because of the mitochondria's negative membrane potential. However, rhod-2 bears one net positive charge only in the AM form. When fully de-esterified, rhod-2 bears three net negative charges which is likely to preclude significant mitochondrial accumulation. The balance of esterase activity in the cytoplasm and mitochondria will determine the major source of the signal from rhod-2 and is likely to contribute to variations in results among studies.

If the small molecule indicators could be selectively targeted to subcellular regions, their speed and wide dynamic range could offer significant advantages over fluorescent protein Ca^2+^ indicators in measuring [Ca^2+^]. Several attempts have been made to target these indicators. In one proof of concept study, peptides which carry organelle-specific import or retention sequences (58) were added to a small synthetic molecule fluorophore (BODIPY). The peptides resulted in the fluorophore being selectively localized to the SR, Golgi, peroxisomes, or nucleus within minutes (58). Although not yet used to target Ca^2+^ indicators, it seems likely that this procedure will prove useful. In an alternative approach, a biarsenical derivative of the small synthetic Ca^2+^ indicator Calcium Green [calcium green FlAsH (CaGF)] has been synthesized that specifically labels fusion proteins that possess four cysteines (tetracysteine tag) (44). This indicator has been genetically targeted to plasma membrane proteins (connexion 43) to measure subplasma membrane [Ca^2+^]. The results showed an uneven distribution and wide variations in the open probability of voltage-dependent Ca^2+^ channels across the plasma membrane and validate the procedure as a way of localizing small synthetic molecule Ca^2+^ indicators to measure fast Ca^2+^ signals (44). In yet another approach which enabled fast small molecule indicators to be targeted, a membrane permeant form of indo-1 which possessed an *O*^6^-benzylguanine side group was synthesized. *O*^6^-benzylguanine specifically interacts with SNAP-tag, an engineered human alkyguanine-DNA alkytransferase. The indicator (indo-1) was localized in cells engineered to express SNAP-tag fusion proteins in the nucleus (59). A fura-2FF Ca^2+^ indicator (a derivative of fura-2 with lower affinity for Ca^2+^ and faster kinetics) also with a *O*^6^-benzylguanine side group has been synthesized to permit subcellular localization again via links to SNAP-tag fusion proteins (60). These procedures enable the spatial specificity of the protein biosensors approach with the fast kinetics (*k*_on_ in the 1–10 × 10^8^ M^−1^ s^−1^ range) and high dynamic range (20–100 fold increase in fluorescence from Ca^2+^-free to Ca^2+^-bound) of the small synthetic indicators and await full experimental exploitation.

## HIGH RESOLUTION IMAGING FOR MEASURING SUBPLASMA MEMBRANE [Ca^2+^]

Rather than targeting the Ca^2+^ indicator to specific regions of the cell, the other major approach to selectively measure Ca^2+^ signals from only certain regions has been to use high resolution imaging methods. Indeed, the desire to image Ca^2+^ at subcellular resolution has been a driving force, which led to significant advances in optics and technology to overcome the constraints imposed on optical microscopy. The main physical limitation imposed by the use of light for high resolution imaging is that of diffraction, which was first fully explained by Ernst Abbe in 1873 (61). Overcoming the “diffraction limit” had been a significant challenge but has become a reality in the last 15 years. Several methods now permit subcellular imaging at resolutions well beyond the diffraction limit. The application of these methods to real biological challenges requires an understanding of the basic physical processes behind the various methods so that the most suitable can be selected.

**Figure 4:**
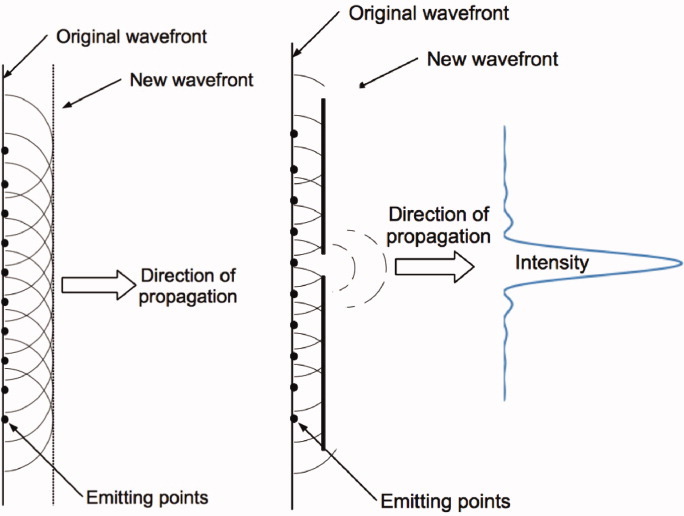
Propagating waves. (A) Shows wave propagating in free space which acts as a series of individual wavelets adding to form the overall light wave (Huygens Wavelets). (B) Shows the effect of an aperture on wave propagation as the wavelets interfere with each other leaving the diffraction intensity pattern shown for a circular aperture. [Color figure can be viewed in the online issue, which is available at wileyonlinelibrary.com.]

Diffraction occurs when a wave meets a physical stop, such as the edges of an aperture. If apertures, including the transmissive part of a lens, were infinite then diffraction would not be the limiting factor (62). At the front of a wave, each point acts as a small emitting source for a new wave, (Fig. 4A) and normally, these small sources add together to produce a uniform wavefront. However, at the edge of an aperture, some of these secondary sources are blocked and some let through and thus light appears to come through the block in certain places, with the positions of these points of light being determined by interference of the light waves (Fig. 4B). A full explanation of this effect can be found in any basic optical text book (63). In an optical microscope, the ability to resolve two closely spaced features in the lateral and axial direction is given by


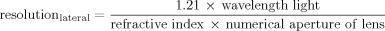



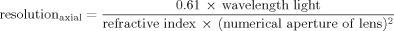


However, the theoretical limit of resolution is difficult to achieve in biological samples. Most samples, even small cells, have a thickness of at least a few microns. As a result of the sample thickness, there will be light from outside the focal plane which will contribute to the image in the focal plane. The light from outside the focal plane will limit the resolution and quality of the image.

Confocal microscopy removes this out of focus light but can only return the image to, at best, the diffraction limit set above. One way to increase the resolution and to overcome the diffraction limit is through the use of fluorescence methods in which either only a single fluorescent molecule is excited (STochastic Optical Reconstruction Microscopy, STORM, or Photo-Activated Localization Microscopy, PALM) (64, 65) or a diffraction limited volume is excited and then most of the fluorescence removed using a second, stimulating light beam, leaving a subdiffraction limited volume to continue fluorescing, before the detector is activated (STimulated Emission Depletion microscopy, STED) (66). More recently, in another approach, a structured illumination (SIM) method has been developed. In this case, a pattern of light is directed onto the sample, and multiple images taken, which, after mathematical treatment, enables improvement of the diffraction limit (67). SIM currently has lead to imaging with a resolution of around 10 nm, that is, ∼30 times less than the diffraction limit. However, each of the above methods is comparatively slow and requires repeated exposures (STORM, PALM, and SIM) or sample scanning (STED). Thus, these methods are not suitable for observing rapid transient events such as the [Ca^2+^] changes in the subplasma membrane space. An alternative method, which provides sub-diffraction limited axial resolution, is total internal reflection fluorescence microscopy (TIRF) (68). In TIRF, the speed limitation is not determined by the excitation beam but only by the speed of the imaging camera and kinetics and brightness of the indicators to enable imaging rates of ∼300 frames per second with adequate signal intensity.

## PRINCIPLES OF TIRF MICROSCOPY

**Figure 5:**
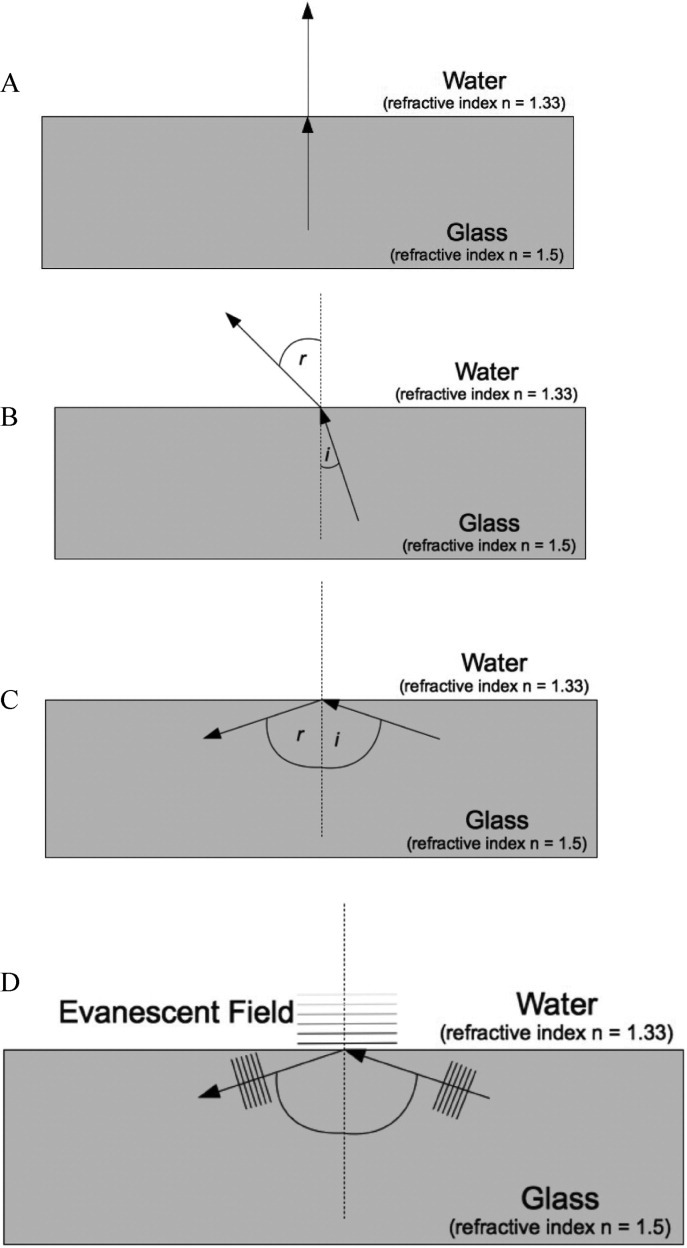
Light, refraction and total internal reflection. (A) Light is undeviated as it passes from high (glass) to low (water) refractive index when at normal incidence. (B) Light bent by refraction (Snell's law) as it passes from high to low refractive indices. (C) Total internal reflection as the angle of incidence exceeds the critical angle. (D) This figure shows the evanescent field propagating from a glass/water interface when angle of incidence exceeds the critical angle. The evanescent field intensity declines exponentially with distance from the interface.

Total internal reflection occurs when light traveling in a material of high refractive index meets a material of lower refractive index (refractive index being a measure of the speed of light in the material). If the light hits the interface at normal incidence (zero degrees), it will pass through undeviated (Fig. 5A). As the angle of incidence increases so the light will be refracted according to Snell's law (Fig. 5B) until a critical angle is reached when the light is totally reflected, that is, the surface acts like a perfect mirror (Fig. 5C). This effect is used in the prisms of binoculars for example and is also the basis of how light can be sent down optical fibers.

Although the light is reflected according to Maxwell's equations (which govern the propagation of light) (63), an electric field is present on the other side of the interface which is known as an evanescent field and has the ability to excite fluorophores (Fig. 5D). In Fig. 5D, a light wave is shown hitting an interface with the light being reflected and the so-called evanescent field propagated across the interface. This field decays rapidly with distance from the interface following an exponential fall off. If a fluorescent molecule is close to the interface, in the evanescent field, then it will be excited. In this way, it is possible to obtain fluorescent images of samples very close to the surface, typically closer than 200 nm, and in certain cases, using a low excitation power, less than 100 nm—significantly better than the diffraction limit. Multiple fluorescent labeling is possible through the use of two excitation fields and different indicators, though care has to be exercised here as the evanescent field created by different wavelengths of light will fall off in slightly different ways. Even though TIRF imaging exceeds the diffraction limit in the axial direction, the limit of resolution in the lateral direction remains at the diffraction limit given above.

**Figure 6:**
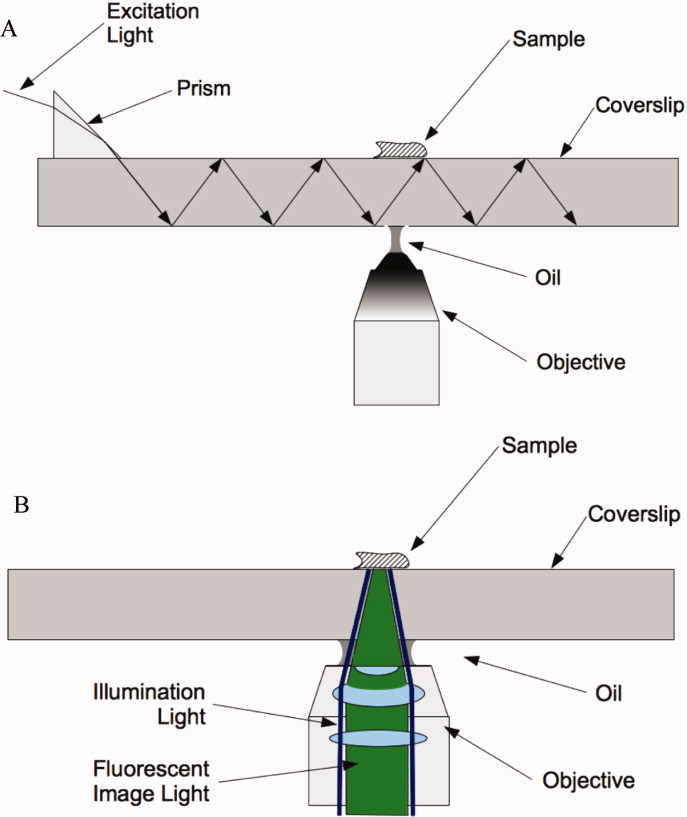
Methods to generate total internal reflection. (A) Prism coupling for TIRF microscopy and (B) TIRF via a very high numerical aperture objective. Excitation light is shown as a dark blue line traveling along the over edge of the objective while the emitted light from the sample fluorophore is shown traveling down through the objective in green.

TIRF, thus, provides a method of selectively exciting molecules close to an interface, and two standard methods have been developed to enable this form of imaging. In the first, a prism is used to direct the light into a glass substrate (Fig. 6A). A collimated beam of light (typically a laser) is directed into the prism and initially the light will pass out of the glass slide. However, as the angle is increased, the light will eventually hit the outer surface of the microscope slide, and undergo total internal reflection and be guided along the slide, or coverslip. An imaging objective observing a short way down field from the prism, can then be used to record fluorescence from samples on the surface being excited by the evanescent field propagating with the light as it makes its way down the slide. Optically, this method works well and enables excellent control of the evanescent field. However, from a practical perspective in a biology laboratory, the exposed optics and laser beams raise laser safety issues, and the prism system requires careful adjustment and repeated alignment to ensure optimal performance which limits usefulness.

An alternative method, and that used by nearly all commercial TIRF microscopes, is the use of a high numerical aperture objective in an epi-illumination configuration (Fig. 6B). In basic terms (specific systems can vary slightly), an annular ring of light is directed into the back aperture of a very high numerical aperture lens (Fig. 6B). The numerical aperture is such that this light is totally internally reflected when it tries to exit the sample slide to create the required evanescent field. The same objective lens then observes fluorescence emitted from the sample in a conventional epi-configuration using the central portion of the lens. The advantage of this method is that the light annulus can be controlled at the rear of the microscope enabling a simple adjustment of the angle of the light to be made to set the TIRF angle through the objective. Thus, no optical coupling (except the normal oil for a high NA objective) is required near the sample, and all the illumination optics can be kept away from the wet (physiological solutions), and less than ideally clean, biological area. In addition, the laser beams can be fully enclosed, reducing the laser safety concerns that exist with exposed beams. Thus, it is clear why this method is preferred by commercial microscope manufacturers. Our system, described below, is a variation on this basic set up and is designed to enable high-speed TIRF imaging and near simultaneous wide-field epi-fluorescence. The method enables measurement of subplasma membrane Ca^2+^ signals and comparison of bulk average [Ca^2+^]_c_(global signals) so that an understanding to the initiation and propagation of the signals is possible.

## OPTICAL INSTRUMENTATION

**Figure 7:**
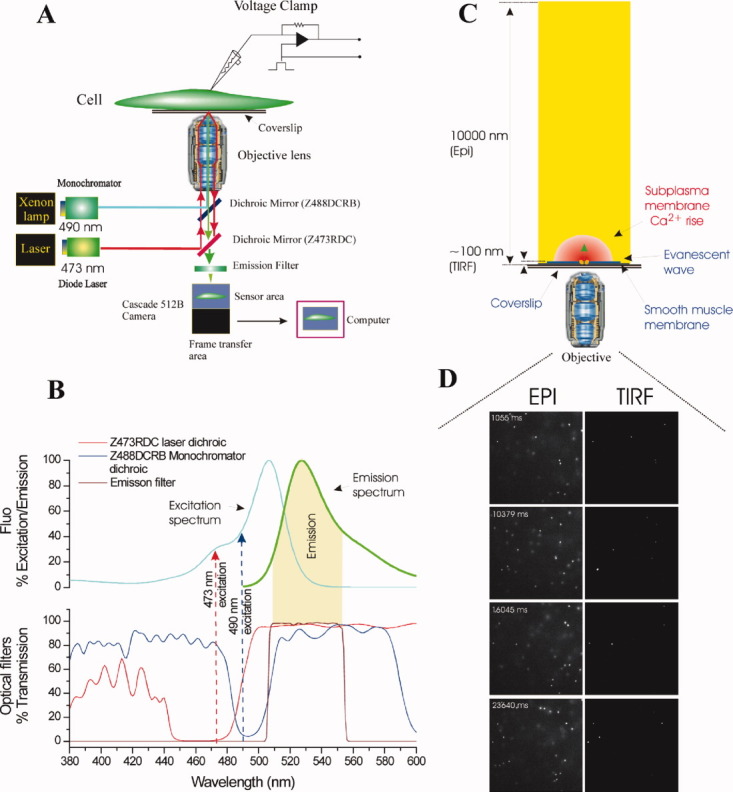
Schematic of the TIRF and wide-field epi-fluorescence system. (A) The experimental system was constructed around a Nikon TE2000U microscope; excitation and emission light paths are shown. Single cells were voltage clamped in whole cell configuration. Cells were loaded with fluo-5F and illuminated by two separate excitation sources (at 490 nm and 473 nm) via a dual epi-illuminator. The first wavelength (490 nm, bandpass 5 nm; blue), was provided by a monochromator and guided via a fiber optic guide through a field stop diaphragm, a neutral density filter and HQ480/40 excitation filter (not shown) before being reflected off a custom-made long-pass dichroic mirror (Z488DCRB). The latter was transmissive in the ranges 380–480 nm and 506–589 nm and reflective from 485 to 499 nm (B). The second excitation wavelength (473 nm; red), for TIRF illumination, was provided by a blue diode pumped laser and guided via a fiber optic coupling through an iris and 10× beam expander. The 473 nm light was then reflected off a dichroic mirror (Z473RDC) and transmitted through the upper dichroic (Z488DCRB) (A and B) and focused to a spot on the back focal plane of the objective lens (Nikon 60×, oil immersion, NA 1.49). The laser focusing lens was mounted on a micrometer driven translation system so that the laser beam could be adjusted to enter the periphery of the objective aperture to achieve total internal reflection at the interface between the cover glass and the aqueous bathing medium. Fluorescence excited in the specimen by the evanescent wave or wide-field epi-illumination was collected through the objective lens, passed through the dichroic mirrors and a barrier filter and imaged by a Phometrics Cascade 512B or evolve 128 cameras (Roper Scientific). The cameras uses a back illuminated frame transfer CCD with on-chip electron multiplication. (C) Enlarged view illustrating the imaging of near membrane Ca^2+^ from the microdomain (red) around a single open channel by evanescent wave (blue) formed by the TIRF objective lens and the simultaneously measured wide-field epi-fluorescence depth of field. The depth of the TIRF field was ∼ 100 nm and that of the wide-field epi-fluorescent field ∼ 10000 nm. (D) Single image frames of sub-resolution (100 nm) fluorescent latex beads floating in solution obtained in TIRF (right) and wide-field epi-fluorescence (left). Under wide-field epi-illumination (EPI, D, left) subresolution fluorescent latex beads floating in solution are illuminated through the field. Individual beads drift through Brownian motion. In TIRF illumination (D,TIRF, right) beads are illuminated only when they are within the evanescent field, that is, within ∼ 100 nm of the coverslip. Beads diffusing in and out of the evanescent field appear and disappear suddenly (Reproduced from ref. 45, with permission from Rockefeller University Press).

The complete optical system is shown in Fig. 7. This system is based upon a conventional Nikon TE2000 inverted microscope and TIRF lens (×60, 1.49NA Nikon Plan Apo). The microscope has two filter turrets for dichroic mirrors on a dual epi-illuminator before the objective lens, enabling both conventional fluorescence excitation and TIRF excitation without moving any components on the microscope. The TIRF light is provided by a frequency-doubled diode-pumped laser operating at 473 nm with a noise of less than 0.01% rms from 1 Hz to 10 MHz. The TEM_00_ output (single spot beam with no mode patterns) was coupled using a home built system into a single-mode optical fiber with a numerical aperture (N.A.) of 0.1 and a core diameter of 3 μm. The laser excitation had ∼1.5 mW of light emerging from the end of the optical fiber. The fiber was connected into the Nikon TIRF attachment at the rear of the TE2000, and the laser excitation was directed to the objective by a custom designed dichroic mirror present in the top turret (Fig. 7A). The objective lens allowed the laser beam to be introduced at the outer edge of the objective aperture to achieve a high angle of incidence of the beam on the coverslip surface. The angle of illumination was adjusted to exceed the critical angle to generate TIRF and an evanescent excitation field giving a penetration ∼100 nm. To determine optimal imaging conditions, a test sample which consisted of subresolution fluorescent beads (170 nm diameter) bathed in fluorescein was imaged. This sample allowed imaging parameters (illumination angle and intensity) which produced maximum image contrast to be determined and an illumination field that was generally even to be generated.

Wide-field epi-fluorescence excitation illumination was provided by an arc lamp with the wavelength controlled via a monochomator to give 490 nm (bandpass 5 nm) coupled via a liquid light guide. The monochromator light was directed to the objective by a custom dichroic on the lower epi-illuminator (Fig. 7A). This light passed through the dichroic in the top epi-illminator and onto the sample.

Rapid (2 ms) switching between TIRF and wide-field illumination was achieved by triggering the laser shutter open using a TTL pulse which simultaneously drove the monochomator to 250 nm (a wavelength that was optically blocked in the system). Terminating the TTL pulse shuttered the laser closed and moved the monochromator to 490 nm.

One unexpected issue arose from the movement of the monochomator between 250 nm and 470 nm. Although the transition only took 2 ms when compared to the CCD cameras frame switch (∼2 μs) in frame transfer mode, it was relatively slow. As a result, a small amount of light initially was recorded during the 2 ms monochromator switch before the final desired wavelength was reached. The light recorded during the monochromator wavelength switch created a slightly increased background fluorescence which limited the ability to resolve of small signals. After exploring several possibilities, the simplest solution was to add a 490 nm narrow bandpass excitation filter to the monochromator excitation light path. Imaging was achieved using EMCCD camera's (a Photometrics Evolve 128 (∼300 frames s^−1^) or Cascade 512B (∼30 frames s^−1^)) (Fig. 7A).

## COMPLICATIONS WITH TIRF

One difficulty which arises when using freshly isolated smooth muscle cells comes from their crenulated structure, which may result in the cells not lying completely flat on the coverslip. The evanescent wave extends only ∼100 nm from the coverslip (Fig. 7C) and if the cells are not flat there is only a small footprint of cell that can be viewed. A major benefit of TIRF, the ability to view channel activity over a large surface of the cell, is restricted by the small imaging footprint. Another issue which may limit full analysis of results is that the Ca^2+^ signals measured in the evanescent field are the sum of signals ranging from saturated fluorophore (near to the channel) to infrequent binding events (a small distance from the channel). Fluorophore saturation will mean the gradient cannot easily be deconvolved so that the fluorescent signal is likely to be an especially nonlinear representation of [Ca^2+^] changes. Even if the dye did not saturate, converting the fluorescent signal to a Ca^2+^ concentration relies on calibration carried out in steady-state conditions. Such conditions are unlikely to be valid in the rapidly changing microdomains under in the subplasma membrane space during Ca^2+^ influx. Indeed it is likely that the assumption that the dye equilibrates instantly with Ca^2+^ will not be correct during influx near a Ca^2+^ channel which is rapid (∼0.6 million ions per second), that is, there is local nonequilibrium within the system (69). Even the rapid small molecule Ca^2+^ indicators are too slow to follow these fast [Ca^2+^] increases. Notwithstanding these difficulties, TIRF is a powerful method to study subplasma membrane events and has generated significant new insights to channel behavior and Ca^2+^ signaling.

## CONCLUSIONS

Subplasma membrane Ca^2+^ signals may be highly localized and extremely rapid events that have a timecourse approximately four orders of magnitude faster than changes in bulk average [Ca^2+^]_c_ (Fig. 1). These subplasma membrane signals target particular responses not only by location but by matching the kinetics of the [Ca^2+^] changes with the speed of the effectors Ca^2+^ binding. Local subplasma membrane Ca^2+^ signals have been studied using targeted Ca^2+^ indicators but the limited dynamic range and slow reaction to changes in [Ca^2+^] may not fully describe the response. Rapid small molecule Ca^2+^ indicators used with high resolution imaging have significantly improved dynamic range and speed but the approach is technically difficult and realistic calibration of signals is not possible. Synthetic indicators that are targeted to particular subcellular sites would combine specificity of the protein biosensors approach with the fast kinetics and high dynamic range of small molecular indicators; these are under development and await full experimental exploitation.
